# A Visit to Philadelphia and New York

**Published:** 1859-11

**Authors:** 


**A Visit to Philadelphia and New York.**

Having been absent from our post some time on a visit to the North, and the labor connected with the October number having fallen upon others, we feel under some obligations to return thanks to (the junior Editor) Dr. W. F. Westmoreland and Dr. H. W. Brown, who have so kindly and efficiently discharged the duties of the Editorial department during our absence; and in accordance with a promise, we also feel that it is due to our readers that some reference should be made to what we saw and heard in a medical way during our sojourn in the "great Medical Centres" of the United States. *Centres* we say, for in the outset we wish it distinctly understood that it is not our purpose to attempt to decide between the conflicting claims of New York and Philadelphia for the title of *the Medical Centre*, both having very great advantages, and though differing somewhat in kind, are in degree, not only fully equal to the demand, but far surpassing any thing that will be used to advantage by the mass of Medical Students. It was our intention to note and enlarge upon, in detail, the various professional matters which came under our observation, but this we found to be impracticable, without undergoing an amount of confinement and labor inconsistent with that leisure which was one of the most important objects of our visit. We have, therefore, left it to the present (except in a few notes) to recall, in a brief and rapid way, some of the more prominent incidents and personages.

Intending to make our *visit* to Philadelphia on the return from New York, our stay here, in going on, was only during the Sabbath and a part of Monday, the latter of which was industriously used in looking around for something professional. For courtesy, kindness and facilities, we were especially indebted to Dr. W. W. Gerhard, who is so well known, not only in our own country, but in Europe, as the assiduous and distinguished devotee to the investigation of Thoracic diseases, who informed me that he would shortly issue a new edition of his valuable work on "Diseases of the Chest," for which we shall be eagerly on the look-out. We were also gratified by a visit to the venerable Professor of the Institutes of Medicine in (our Alma Mater) the University of Pennsylvania, Dr. Samuel Jackson, whose bright intellect seems to be undiminished in lustre by the flight of years, but whose locomotive powers have gradually declined, until it is with great difficulty he can leave his house or undergo any active duties. It has rarely been our fortune to spend a more pleasant and profitable hour than that with which we were honored by this "old man eloquent," in whom the fires of mental youth and energy are yet burning brightly, notwithstanding the distressing physical infirmities under which he has so long labored. He still continues his labors in the University, and seems to be very unwilling to be regarded as an "Old Fogy," but is still determined to keep pace, not only with "Young America," but progressive Medical Science everywhere. We had also, while in Philadelphia, upon this occasion, an interesting visit to the Pennsylvania Hospital, where we had an opportunity of accompanying in his round of the wards, Dr. J. F. Meigs, the author of the work on the Diseases of Children, and the son of the distinguished Georgia Professor of Obstetrics in the Jefferson Medical College, Dr. Chas. D. Meigs. Dr. J. F. Meigs was the attending physician at the time of our visit, and extended courteous attentions for which we feel indebted, and exhibited quite a variety of disease, to which, however, our plan will not allow us to refer in detail.

As before remarked, intending to make some stay in Philadelphia upon our return, we passed on to New York, with the Medical men and advantages of which, we were much less familiar, where the first medical man we met was Dr. J. Marion Sims, formerly of Montgomery, Alabama, and now of the "Woman's Hospital," New York, whose name is eminently known throughout the Medical world in connection with the relief of that most distressing malady, Vesico Vaginal Fistula.

Such is our admiration of Sims, and our high appreciation of his labors in behalf of the profession and of suffering humanity, that we scarcely know how to speak of him. It is, however, certain, that there are few medical men, either in this country or elsewhere, with whom we would sooner exchange positions, or to whom, in our judgment, the profession is more indebted; and if he has vanity (though we saw less of it in him than is usual in distinguished medical or other men, or than he is fully entitled to) with which he has been charged by those who would rob him of his well-earned fame, in our judgment it is scarcely a spot upon the sun of his bright and glowing qualities of head and heart.

We care not if he has claimed too much for himself in connection with "Silver Sutures in Surgery," we shall raise our feeble voice in his favor, believing, as we do, that in the aggregate, his labors and contributions entitle him not only to the *unqualified* commendation, but the gratitude of the profession; and so far from having shown a disposition to thrust his own merits or claims before the public, his course has been characterized by a remarkable degree of *modesty* and *forbearance;* and altogether, a more genial, courteous, unpretending and deserving gentleman we do not know in our ranks, than Dr. J. Marion Sims. We are glad to know that he is reaping the reward of his persevering and self-sacrificing labors, and that there is an almost certain prospect of the realization of what now seems to be the highest object of his ambition: the establishment of a Woman's Hospital upon a magnificent scale, of which the present institution is regarded as the mere nucleus. This gentleman is devoting himself exclusively to the treatment of diseases peculiar to females, and gave us opportunities of witnessing his plans of treatment, for which we feel greatly indebted. He seems to attach very considerable importance to a division of the os and neck of the uterus, in that form of dysmenorrhœa dependent upon or connected with a fibrous condition of that portion of the organ; and to arrest the hemorrhage, which is sometimes very considerable, Dr. Sims uses the per sulphate of Iron, which he thinks greatly preferable to the per chloride, and with the use of this, he does not seem to apprehend any danger, whatever, from hemorrhage or any interference from it with the process of healing. He is now operating constantly upon the most unfavorable cases of Vesico Vaginal Fistula sent to him by surgeons from various parts of the country—the cases which are the most readily relieved being operated upon by those under whose charge they first come. I also saw at the "Woman's Hospital," the commencement of an operation (which particularly attracted my attention) for the formation of a new urethra which had been lost by sloughing, in connection with which in the same case there was present both a Vesico and Recto Vaginal Fistula, which Dr. Sims proposed to close, a case he remarked, that he would not have undertaken, but for an operation which he had performed, to a great degree successful, in a somewhat similar case before. In this connection we would state that in a conversation held with a friend in Richmond, Va., a prominent medical gentleman of that city, upon our return, in reference to the remarkable success of Sims in operating for Vesico Vaginal Fistula, he expressed surprise that they could not attain any such successful results in Richmond, and seemed to be somewhat incredulous in regard to the reported success elsewhere—he stated that their operations closed the Fistula temporarily, but the adhesions frequently gave way afterwards. I have heard the same difficulty suggested elsewhere. Having no doubt of the entire success of Sims' operations, we suppose from what we saw and heard of his procedure, that his greater success is owing not simply to his greater experience, but also, to his greater patience and perseverance, and to his not attempting to accomplishing too much at a single operation—not being discouraged by any amount of failures, and not discharging the patient until the permanent cure is beyond a question. But we have probably said enough upon this point; perhaps more than prudence would dictate, and yet not so much as our own feelings would prompt.

We have yet to notice a variety of other medical matters in New York and Philadelphia, and to express our appreciation of, and gratitude for, great kindness and favors received at the hands of a number of professional brethren of both cities. We shall recur to the subject in the next number of the Journal.

